# Functional neuroimaging with default mode network regions distinguishes PTSD from TBI in a military veteran population

**DOI:** 10.1007/s11682-015-9385-5

**Published:** 2015-04-23

**Authors:** Cyrus A. Raji, Kristen Willeumier, Derek Taylor, Robert Tarzwell, Andrew Newberg, Theodore A. Henderson, Daniel G. Amen

**Affiliations:** Department of Radiology, UCLA Medical Center, 757 Westwood Blvd, Los Angeles, CA 90095 USA; Department of Research, Amen Clinics, Inc, Costa Mesa, CA USA; Faculty of Medicine, Department of Psychiatry, University of British Columbia School of Medicine and Clinical Director of Research for Mental Health, Lions Gate Hospital, Vancouver, BC Canada; Department of Radiology, Thomas Jefferson University, Philadelphia, PA USA; The Synaptic Space and The International Society of Applied Neuroimaging, Denver, CO USA

**Keywords:** Functional Neuroimaging, PTSD, SPECT, TBI

## Abstract

PTSD and TBI are two common conditions in veteran populations that can be difficult to distinguish clinically. The default mode network (DMN) is abnormal in a multitude of neurological and psychiatric disorders. We hypothesize that brain perfusion SPECT can be applied to diagnostically separate PTSD from TBI reliably in a veteran cohort using DMN regions. A group of 196 veterans (36 with PTSD, 115 with TBI, 45 with PTSD/TBI) were selected from a large multi-site population cohort of individuals with psychiatric disease. Inclusion criteria were peacetime or wartime veterans regardless of branch of service and included those for whom the traumatic brain injury was not service related. SPECT imaging was performed on this group both at rest and during a concentration task. These measures, as well as the baseline-concentration difference, were then inputted from DMN regions into separate binary logistic regression models controlling for age, gender, race, clinic site, co-morbid psychiatric diseases, TBI severity, whether or not the TBI was service related, and branch of armed service. Predicted probabilities were then inputted into a receiver operating characteristic analysis to compute sensitivity, specificity, and accuracy. Compared to PSTD, persons with TBI were older, male, and had higher rates of bipolar and major depressive disorder (*p* < 0.05). Baseline quantitative regions with SPECT separated PTSD from TBI in the veterans with 92 % sensitivity, 85 % specificity, and 94 % accuracy. With concentration scans, there was 85 % sensitivity, 83 % specificity and 89 % accuracy. Baseline-concentration (the difference metric between the two scans) scans were 85 % sensitivity, 80 % specificity, and 87 % accuracy. In separating TBI from PTSD/TBI visual readings of baseline scans had 85 % sensitivity, 81 % specificity, and 83 % accuracy. Concentration scans had 80 % sensitivity, 65 % specificity, and 79 % accuracy. Baseline-concentration scans had 82 % sensitivity, 69 % specificity, and 81 % accuracy. For separating PTSD from PTSD/TBI baseline scans had 87 % sensitivity, 83 % specificity, and 92 % accuracy. Concentration scans had 91 % sensitivity, 76 % specificity, and 88 % accuracy. Baseline-concentration scans had 84 % sensitivity, 64 % specificity, and 85 % accuracy. This study demonstrates the ability to separate PTSD and TBI from each other in a veteran population using functional neuroimaging.

## Introduction

Traumatic brain injury (TBI), whether by direct impact or due to blast wave effects, is an all-too-common and devastating feature of armed conflict. With the proliferation of improvised explosive devices (Okie 2005), TBI became the “signature wound” of the conflicts in Afghanistan and Iraq (Snell and Halter 2010). From these conflicts, returning soldiers also report experiencing severe psychological trauma with 50 % witnessing the death or injury of a friend, 10 % had been injured themselves, and over 19 % had symptoms consistent with posttraumatic stress disorder (PTSD) (Tanielian et al. 2008). Another study suggests that 90 % of soldiers witnessed a traumatic episode while in service (Hoge et al. 2008). It is therefore not surprising that 400,000 military personnel and veterans have been diagnosed with PTSD or TBI since 2001, and many have been diagnosed with both (Congressional Budget Office 2012).

TBI and PTSD are both common in military populations, making it a challenge to distinguish the two conditions from one another (Hoge et al. 2008). Clinically, these populations may overlap by 33 to 42 % (Lew 2005). A recent study in Veterans Administration facilities revealed 73 % of patients reporting TBI were comorbid for PTSD (Taylor et al. 2012), while 13.5 % of military personnel from recent conflicts reported PTSD (Dursa et al. 2014). For patients who have both TBI and PTSD, which generate many of the same symptoms, the VA acknowledges that the patient is often diagnosed with one or the other. Note that several of the question items within the Clinician-Administered PTSD scale (Blake et al. 1998) can be symptoms of PTSD, such as poor concentration, memory difficulties, anhedonia, social isolation, sleep difficulties, and irritability. The VA recently concluded there was a lack of diagnostic accuracy for the dually affected veteran (Congressional Budget Office 2012). Assessment of an event can be further confounded because memories of traumatic experiences, particularly long after the actual event occurred, can be inconsistent. Lastly, there is often a mismatch between performance on neuropsychological testing and the patient’s experience of the deficits in those with mild TBI, which is the most common form of TBI (Brenner et al. 2010). Some evidence suggests that additional recruitment of cortical areas during a task contributes to performance on neuropsychological testing in those with TBI (Van Boven et al. 2009).

The currently available treatments of PTSD and TBI are different. Moreover, the treatments for PTSD may be harmful or, at best, not helpful in the case of TBI. While the well-established PTSD treatments of exposure therapy (Rauch et al. 2012; Schnyder 2014), cognitive processing therapy (Walter et al. 2014) and trauma-focused cognitive behavioral therapy (Schnyder 2014) are helpful in PTSD, there is no evidence to suggest that they would be helpful for those with mild TBI who are misdiagnosed as having PTSD. The pharmacological treatments for PTSD include the serotonin reuptake inhibitors, serotonin-norepinephrine reuptake inhibitors, benzodiazepines, mood stabilizers, and atypical antipsychotics (Jain et al. 2012; Watts et al. 2013). While antidepressants can be useful in managing some of the symptoms of TBI, the prescribing of benzodiazepines to those with TBI can impede function or be dangerous (Silver et al. 2011; Kennedy et al. 2007; Morgan et al. 2012). Similarly, antipsychotics are often prescribed for PTSD (Morgan et al. 2012) and clinical studies support the use of atypical antipsychotics (Wang et al. 2013; Hermes et al. 2014; Krystal et al. 2011); however, antipsychotics have been shown to impede recovery or be dangerous in clinical studies and animal models of TBI (Kline et al. 2008; Phelps et al. 2014). Curiously, having a diagnosis of TBI increases the likelihood that a veteran will be prescribed a benzodiazepine or an antipsychotic. While 41 % of veterans with PTSD were prescribed benzodiazepines, those with both PTSD and TBI had a 67 % chance of being prescribed a benzodiazepine. Antipsychotics were prescribed to 25 % of veterans with PTSD, but 40 % of those with both PTSD and TBI (Morgan et al. 2012). Other treatments for PTSD, such as transcranial magnetic stimulation (Berlim and Van Den Eynde 2014), can be dangerous in TBI due to induction of seizures (Castel-Lacanal et al. 2014). Emerging treatments for TBI are more targeted and require an understanding of what portion of the individual’s brain is involved, while there are evidence-based, well-established treatments for PTSD (Ursano et al. 2004). Therefore, where clinical uncertainty exists, an accurate biomarker for each of these conditions may be helpful in providing diagnostic clarity that would then allow initiation of an appropriate treatment algorithm.

The default mode network (DMN), consisting of the inferior orbital frontal cortex, anterior cingulate gyrus, posterior cingulate gyrus, hippocampus, precuneus, superior parietal lobe, and angular gyrus (Buckner et al. 2008), shows hyperperfusion in patients diagnosed with PTSD and hypoperfusion in patients diagnosed with TBI (Erickson et al. 2014; Dubroff and Newberg 2008; Newberg and Alavi 2003; Newberg et al. 2014; Liu et al. 2013). By further elucidating the correlation between increased perfusion in DMN structures in PTSD and decreased perfusion in TBI, clinicians may be able to more reliably diagnose PTSD from TBI.

Brain single photon emission computed tomography (SPECT) is a widely available functional neuroimaging study indicated for the evaluation of TBI in the absence of anatomical findings (Davis et al. 2000). A recent review of three decades of research (Raji et al. 2014) supports the diagnostic utility of SPECT in TBI. The purpose of this study was to assess the DMN using quantitative SPECT to assess whether PTSD can be separated from TBI in a veteran population. In particular, brain regions associated with DMN were analyzed using SPECT to determine whether there were significant differences between individuals diagnosed with PTSD and those diagnosed with TBI. Because previous literature suggests that PTSD patients have increased perfusion of DMN (Philip et al. 2014; Shin et al. 1999), while TBI patients have decreased perfusion of DMN, DMN perfusion SPECT data could act as a biomarker in the differentiation of the two diseases. Therefore, brain perfusion SPECT could provide, in cases of clinical uncertainty, an accurate diagnosis and guide patients with PTSD or TBI toward appropriate treatment by examining the status of DMN perfusion in the baseline state.

## Methods

### Study subjects

All subjects were obtained for retrospective analysis from a large multisite psychiatric database involving 20,746 patients who came for evaluation of complex, treatment resistant issues to one of nine outpatient clinics (Newport Beach, Costa Mesa, Fairfield, and Brisbane, CA, Tacoma and Bellevue, WA, Reston, VA, Atlanta, GA and New York, NY) between 1995 and 2014. Diagnoses were made by board certified or eligible psychiatrists, using all of the data available to them, including detailed clinical history, mental status examination and DSM-IV or V criteria, consistent with the current standard of care. Anonymous data was mined from a research database containing SPECT data and corresponding clinical information (IRB #004). This retrospective review was approved by an accredited institutional review board IntegReview (http://www.integreview.com/). Specifically, peacetime or combat veterans with either a DSM-IV diagnosis of PSTD, TBI, or those with PTSD and TBI were selected for this analysis. These inclusion and exclusion criteria yielded a total of 196 subjects (115 TBI, 36 PTSD, and 45 with PTSD and TBI). Subject characteristics are displayed in Table 1. All individuals in this study were right handed. No subjects with epilepsy, a condition that can produce dramatic focal SPECT abnormalities, were included in the analysis. Subjects with history of stroke were also excluded from the study. No sedating or stimulating drugs such as caffeine were consumed on the day of the SPECT examination per Society for Nuclear Medicine scanning guidelines for SPECT scanning (Juni et al. 1998, 2009; Kapucu et al. 2009).

**Table 1:** Subject characteristics

Variable	PTSD (*n* = 36)	TBI (*n* = 115)	TBI/PTSD (*n* = 45)	TBI compared to PTSD; PTSD/TBI compared to PTSD or TBI (χ or t, *p*-value)
Age	45.8 ± 15.4	38.6 ± 12	42 ± 14.4	−2.5, 0.01; −0.8, 0.4
Gender %Male (n)	69 (25)	94 (108)	78 (35)	15, < 0.001; 3.1, 0.09
Race % Caucasian (n)	75 (27)	77 (89)	84 (38)	0.35, 0.49; 4.8, 0.7
Dementia% (n)	3 (1)	5.2 (6)	4 (2)	0.09, 0.67; 0.00, 1
Depression% (n)	33 (12)	50 (58)	36 (16)	3.7, 0.04; 0.02, 0.87
Schizophrenia% (n)	3 (1)	2.8 (3)	0 (0)	0.17, 0.56; 1.8, 0.34
ADHD% (n)	70 (25)	56 (64)	71 (32)	2.4, 0.09; 0.34, 0.55
Bipolar% (n)	3 (1)	14 (16)	11 (5)	5.3, 0.04; 1.4, 0.25
Substance Abuse% (n)	25 (9)	17 (20)	27 (12)	0.74, 0.27; 0.48, 0.54
Alcohol Use% (n)	14 (5)	6 (7)	13 (6)	1.2, 0.36; 0.27, 0.59
Location %Newport (n)	36 (13)	39 (45)	36 (16)	0.106, 0.84; 5.2, 0.22

Among the TBI group, 53 % had mild TBI, 4.3 % had moderate TBI, and 3.5 % had severe TBI. There were 38.3 % who had an unknown severity of TBI. Psychiatric comorbidity was similar to other populations of veterans with complex issues (Bowe and Rosenheck 2015). With respect to the veteran demographics by service, 41 % were Army, 17 % were Marines, 19 % were Air Force, and 23 % were Navy. Of the subjects with TBI, 19 % were associated with an event that occurred during service.

### SPECT imaging

SPECT was obtained in conjunction with clinical assessments before any intervening treatment. SPECT was performed as previously described (Amen et al. 2008a, b). For each procedure, an age- and weight-appropriate dose of technetium-99m exametazime was administered intravenously at rest and while performing a concentration task. For the baseline scans, patients were injected while they sat in a dimly lit room with eyes open. Patients were scanned approximately 30 min after injection. For the concentration scans, patients were injected three minutes after starting the Conners Continuous Performance Test (C-CPT). Approximately 30 min after the injection, subjects were scanned. Photon emission was captured using a high resolution Picker (Phillips) Prism XP 3000 triple-headed gamma camera with fan beam collimators with data collected in 128x128 matrices, yielding 120 images per scan with each image separated by three degrees spanning 360 degrees. A low pass filter was applied with a high cutoff. Chang attenuation correction was performed (Chang 1977, 1984). Transaxial slices oriented horizontal to the AC-PC line were created along with coronal and sagittal images (6.6 mm apart, unsmoothed). Three dimensional reformats were generated for review based on Odyssey image visualization software.

#### SPECT region of interest analysis

Bilateral ROI counts were derived from the anatomical regions in the Automated Anatomical Labeling (AAL) atlas (Tzourio-Mazoyer et al. 2002). These quantitative ROI metrics were in no way used to aid in the clinical diagnosis of PTSD or TBI. To account for outliers, T-score derived ROI count measurements were derived using trimmed means (Thomas and Tilanus^1964^) that are calculated using all scores within the 98 % confidence interval (−2.58 < Z < 2.58). The ROI mean for each subject and the trimmed mean for the sample are used to calculate T with the following formula: T = 10*((subject ROI_mean – trimmed regional_avg)/trimmed regional_stdev) + 50.

#### Statistical analyses

All analyses were performed using Statistical Package for Social Science (SPSS, version 22, IBM, Armonk, NY). Multiple imputations identified less than 10 % missing data in the analysis. Default mode network regions as defined (Raichle et al. 2001; Buckner et al. 2008) were inputted into a binary logistic regression analysis controlling for co-morbidities and demographics as defined in Table 1 and also controlling for TBI severity and armed service branch. The default mode network regions selected were: bilateral angular gyrus, anterior and posterior cingulate gyri, the precuneus, inferior orbital frontal cortex, superior parietal lobe, hippocampus, and parahippocampal gyrus. Both baseline and concentration regions were selected for separate binary logistic regression analyses. Additionally, the difference between baseline and concentration regions was also inputted into a separate analysis. For each analysis, predicted probabilities were extracted from this analysis and then inputted into a receiver operating characteristic curve to calculate sensitivity (the true positive results as a fraction of the true positives plus false negatives), specificity (the true negative results as a fraction of false positives plus true negatives), and accuracy (the area under the curve defined as true positives plus true negatives divided by total sample).

## Results

Quantitative SPECT imaging of DMN regions, at baseline or on concentration task, distinguished veterans with PTSD from those with TBI with an accuracy range of 87–94 %. These measures can delineate TBI from those with PTSD and TBI with an accuracy range of 79–83 %. For distinguishing veterans with PTSD from those with both, the accuracy range is 85–92 %.

Table 2 details the diagnostic utility of baseline, concentration, and baseline-concentration default mode network regions in distinguishing TBI from PTSD:

**Table 2:** Diagnostic utility of quantitative brain SPECT default mode network

Variable	Sensitivity	Specificity	Accuracy (95 % CI)
PTSD from TBI			
Baseline	92	85	94 (88–99)
Concentration	85	83	89 (83–95)
Baseline-Concentration	85	80	87 (79–95)
PTSD/TBI from TBI			
Baseline	85	81	83 (76–90)
Concentration	80	65	79 (81–88)
Baseline-Concentration	82	69	81 (74–88)
PTSD/TBI from PTSD			
BL	87	83	92 (86–99)
Conc	91	76	88 (81–96)
BL-Conc	84	64	85 (75–94)

**Fig. 1:**
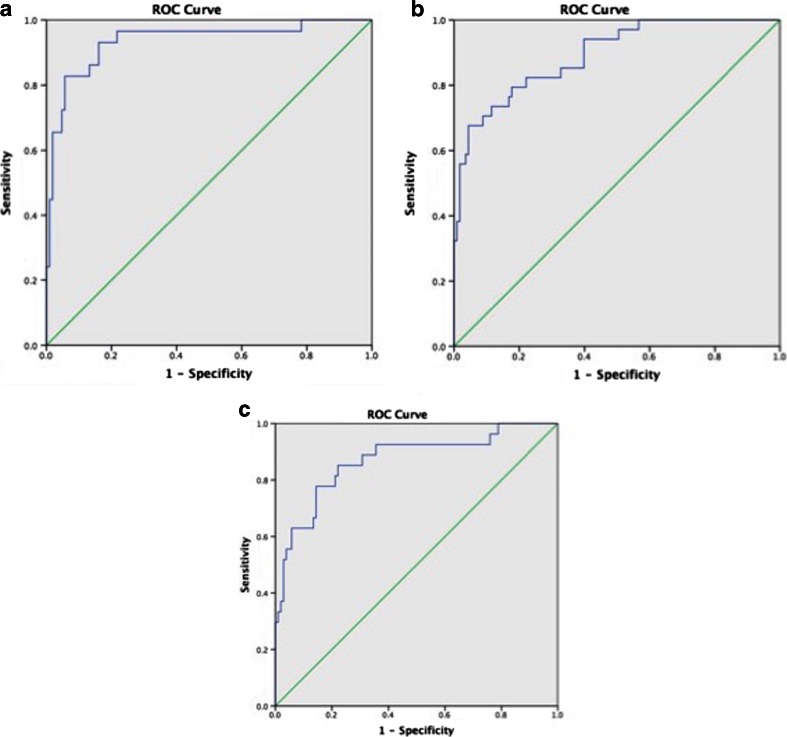
Receiver operating characteristic curves for quantitative baseline (**a**), concentration (**b**) and (Baseline-Concentration) (**c**) default mode network region in separating PTSD from TBI

**Fig. 2:**
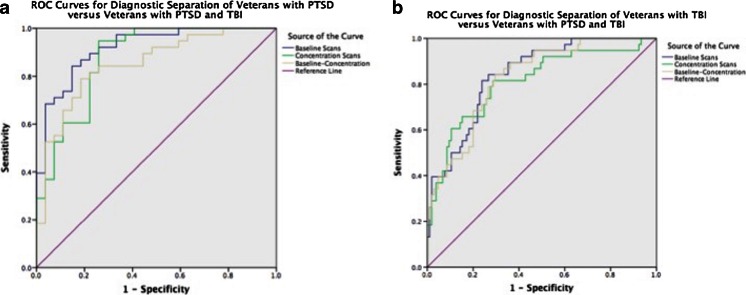
**a** Shows ROC curves delineating veterans with PTSD from those with both PTSD and TBI. **b** Displays ROC curves distinguishing veterans with TBI from those with PTSD and TBI

**Fig. 3:**
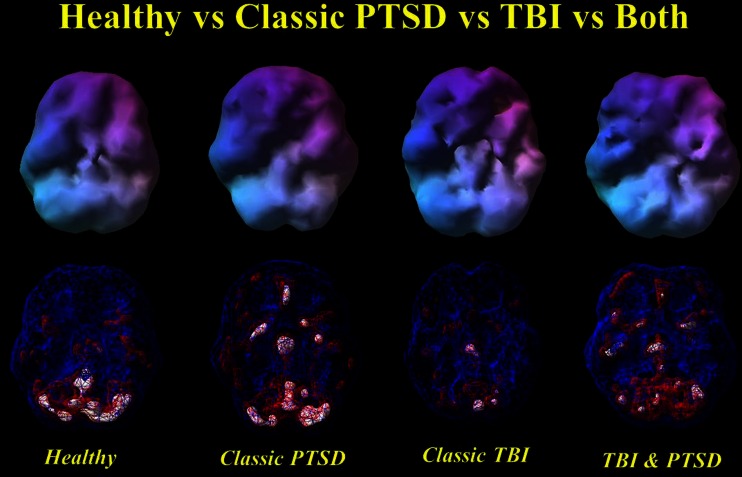
Healthy vs Classic PTSD vs TBI vs Both

Figure 1 shows a summary of the diagnostic utility of brain SPECT in distinguishing PTSD from TBI. Figure 2 shows two groups of ROC curves. Figure 2a shows ROC curves delineating veterans with PTSD from those with both PTSD and TBI. Figure 2b displays ROC curves distinguishing veterans with TBI from those with PTSD and TBI. Figure 3 shows two rows of volume rendered SPECT images, each from separate subjects. The first volume rendered row shows inferior underside surface rendered images. The second row shows intensity projection images in which white colors represent the top 8 % and red colors represent the top 15 % of cerebral flow in that subject’s brain compared to their whole brain perfusion. A healthy control shows normal higher perfusion to the cerebellum. The PTSD subject shows increased perfusion in the brain – particularly in the frontal lobes. The TBI subject shows decreased perfusion throughout by comparison. The subject with both PTSD and TBI shows perfusion that is intermediate in that it is lower than the person with PTSD but higher than the subject with TBI. These representative images highlight how, in a given set of subjects, there is increased perfusion in PTSD and hypoperfusion in TBI with an intermediate imaging pattern in persons with both.

## Discussion

This study identified regions that distinguished TBI patients from those with PTSD. Because quantitative ROIs were not used in any way to initially establish the clinical diagnoses of TBI and PTSD, they serve as a particularly rigorous independent predictor of diagnostic category. Use of the DMN for discriminating TBI from PTSD in baseline versus concentration states further expands upon the evolving concept of applying these regions in better understanding brain health and disease (Raichle et al. 2001; Morcom and Fletcher 2007). SPECT imaging can help distinguish PTSD and TBI with good sensitivity and specificity on both baseline and concentration scans. These data demonstrate that SPECT may be a reasonably sensitive test, with accuracy in a clinically relevant range for distinguishing TBI from PTSD in veterans. SPECT data in the study show that elements of the DMN are affected in both TBI and PTSD, but in an opposite manner. Specifically, the DMN is hyperperfused in individuals diagnosed with PTSD and hypoperfused in patients diagnosed with TBI. The data suggests that a specific neural network, the DMN, is implicated in both PTSD and TBI contributing to the similarity in symptoms. However, the pathophysiologies of the diseases are different in the way they effect perfusion of DMN. SPECT may therefore allow clinicians to differentially diagnose PTSD and TBI. While the data suggest that baseline SPECT alone may be sufficient for delineation of PTSD versus TBI, concentration SPECT may be useful in further refining the neurophysiological deficits in persons for which their TBI or PTSD may manifest with hypo or hyper-attentive symptoms. Given the wide availability of brain SPECT imaging and the need for accurate diagnosis in distinguishing PTSD from TBI, if this test retains accuracy in prospective trials, it could be translated into the clinical care of veteran population.

Peterson’s recent survey takes a network-based approach to findings in 11 fMRI studies, which met her quality threshold over the survey period, 2009 to mid-2013 (Peterson et al. 2014). They report a positive correlation between DMN activity in PTSD severity in five studies, negative in two, suggesting that DMN overactivity may be responsible for symptoms associated with PTSD. DMN activity and PTSD symptom severity have been shown to correlate positively (Lanius et al. 2010) while DMN activity and TBI have been shown to correlate negatively (Bonnelle et al. 2011). DMN is activated when individuals are performing internally focused tasks, which include the recall of autobiographical memories, the consideration of the thoughts and feelings of others, and attention attenuation (Sandrone and Bacigaluppi 2012). This data further corroborates the existing evidence on DMN alterations that differentially influence persons with PTSD versus TBI. Taken together, the results of this study suggest that DMN SPECT perfusion data may act as a reasonably accurate biomarker to differentially diagnose PTSD and TBI as has been done in other conditions such as dementia (Henderson^2012^).

A subtle, but critical, aspect of neuroimaging is the ability to show the patient a picture of what is occuring in their brain. Only one-quarter to one-third of soldiers and veterans with PTSD who screened positive for PTSD receive treatment (Hoge et al. 2014; Stecker et al. 2013, 2014). Guilt, shame, fears of medications, lack of confidence in the therapies offered, and denial of a medical problem are among the reasons treatment is avoided even though efficacious medications and psychotherapy are available for PTSD and correct diagnosis will facilitate use of evidence-based practices (Hoge et al. 2014; Stecker et al. 2013, 2014; Murphy et al. 2014; Bryan et al. 2013). The barriers to treatment for patients with TBI are unknown, but can be suspected to be similar. Herein, we show that the functional brain scan can provide a depiction of the biological processes underlying a patient’s symptoms, which can reduce stigma, foster efforts to seek a medical solution, and remove self-blame/shame.

Advantages of this study include the application of a quantitative functional neuroimaging methodology to a well-characterized veteran cohort. An additional advantage was a multivariable approach in the logistic modeling that controlled for multiple other factors including psychiatric co-morbidities. The main caveat of this study is its retrospective nature. Future work will benefit from a prospective approach to better understand how imaging data may improve patient outcomes. Functional neuroimaging with SPECT can be used to differentiate PTSD and TBI by examining the activity of DMN in the baseline state. By examining the difference in perfusion of DMN using SPECT, veterans can be better assessed for the purpose of diagnostically separating TBI from PTSD. Given the wide availability of SPECT and the existence of internationally accepted standards in nuclear medicine for performing baseline state perfusion scans (Kapucu et al. 2009; Juni et al. 2009), the potential for rapid clinical translation makes this test especially significant in the clinical care of persons with PTSD or TBI.
